# vWDI is inherited in an autosomal dominant manner with incomplete penetrance, in the Kromfohrländer breed

**DOI:** 10.1186/s40575-019-0073-4

**Published:** 2019-05-16

**Authors:** Julia H. Segert, Jana-Marie Seidel, Walter J. Wurzer, Anja M. Geretschlaeger

**Affiliations:** 1 FERAGEN GmbH, Strubergasse 26, 5020 Salzburg, Austria; 2 Speciality Pharma Service Austria e.U, Altenmarkt/Triesting, Austria

**Keywords:** Von Willebrand disease, Von Willebrand factor, Genetic disorder, Genetic testing, Kromfohrländer

## Abstract

**Background:**

Von Willebrand disorder type I (vWDI) is known as an inherited bleeding disorder in different dog breeds following an autosomal recessive inheritance. The Kromfohrländer is a rare dog breed with an increased incidence of unclear bleeding episodes and prolonged coagulation time during/after surgery or injuries, indicating a defect in one or more critical proteins of the coagulation cascade.

**Objective:**

The objective of this study was to determine whether the c.7437G > A mutation in the *VWF* gene previously shown to cause von Willebrand disorder type I in Doberman Pinscher is also linked to this disease in the Kromfohrländer breed and to serum concentrations of vWF. Furthermore, establish a possible link between bleeding phenotype, vWF serum concentrations and *VWF* mutation status.

**Results:**

Eighty-seven Kromfohrländer were genotyped for the G > A von Willebrand type I mutation. For detection of the associated mutation we used an endpoint genotyping method. We identified the G > A von Willebrand type I mutation in 80.5% of our study population. 65.5% were heterozygous (WT/MUT) and 15.0% were homozygous for the mutation (MUT/MUT). 21% of the overall study population exhibited bleeding symptoms. 45.5% of all homozygous dogs (MUT/MUT) showed bleeding symptoms. In contrast, wild-type homozygotes exhibited no bleeding symptoms, whereas 23.2% of the heterozygotes did. VWF serum concentrations varied from 28 to 137% in wild-type dogs while in heterozygous and homozygous dogs the concentration ranged from 3 to 77% and 1 to 23%, respectively (*p* < 0.05)

**Conclusion:**

Based on our data, we found the G > A mutation in the *VWF* gene in the Kromfohrländer breed and the subsequent vWDI as the underlying cause for the bleeding episodes and delayed coagulation in heterozygous and homozygous dogs. Since both, heterozygotes and homozygotes show reduced vWF serum concentrations and exhibit to a certain percentage the vWD syndrome phenotype, we postulate that, in contrast to most other vWDI affected breeds, inheritance follows an autosomal dominant mode with incomplete penetrance.

## Plain english summary

A variety of bleeding diseases are known in dogs including von Willebrand disease type I. This genetic disorder affects the von Willebrand factor in plasma which is important for blood clotting. A genetic mutation known from the Doberman Pinscher was also found in other dog breeds including the Kromfohrländer. Inheritance of the disorder is described in most breeds as autosomal recessive trait resulting in mild bleeding symptoms.

The Kromfohrländer is an especially rare breed based on two founders which was established in the years after World War II in Germany. Reports on excessive bleeding during surgeries, birth or neutering/spaying are well known but without any clear association to a von Willebrand bleeding disorder. For this reason we were interested in finding a link between the von Willebrand disease type I mutation and a reduced plasma von Willebrand factor leading to bleeding symptoms in this breed

Genetic testing of Kromfohrländer for von Willebrand type I mutation revealed a high number of heterozygous dogs for the mutation. Determination of the plasma factor showed that dogs heterozygous and homozygous for the vWDI mutation have a clearly reduced plasma level compared to wild-type dogs. This increases the risk for developing symptoms of a blood coagulation disorder due to the reduced von Willebrand factor concentrations.

In most breeds, inheritance of von Willebrand disease type I is described as an autosomal recessive trait. In the Kromfohrländer breed, symptoms of bleeding were not only observed in homozygous but also in heterozygous dogs. Based on this finding, heredity of von Willebrand Disease type I in the Kromfohrländer follows an autosomal dominant inheritance with incomplete penetrance resulting in severe bleeding symptoms in the Kromfohrländer breed. To avoid further distribution of the mutation within the population it would be of importance to perform genetic testing with a long term breeding program for reducing the number of heterozygous and homozygous dogs without an additional reduction of the genetic diversity.

## Background

The von Willebrand factor (vWF) is a large multimeric plasma glycoprotein required for platelet adhesion and aggregation. These multimers vary in size and circulate in the blood plasma. A deficiency or defective vWF results in von Willebrand disease (vWD). In dogs and in humans vWD is one of the most common inherited bleeding disorders [1, 2]. vWD can be classified in 3 different types based on the clinical severity and quantity of von Willebrand factor. Type I is the most common type of vWDI in dogs and accounts for more than 95% of all cases, meaning that nearly all canine cases can be classified as a type I disorder [3]. This type is characterized by low plasma vWF concentrations responsible for mild to moderate bleeding symptoms. Patients with a type II disorder have variably reduced vWF in plasma with an abnormal multimeric structure resulting in moderate to severe bleeding. This type of vWD is rare in dogs and to date vWD type II has been only described for German Shorthaired Pointers and German Wirehaired Pointers [4, 5]. A novel *VWF* variant associated with vWD type II in German Shorthaired and German Wirehaired Pointers was reported in 2017 by Vos-Loohuis et al. [6]. This second variant possibly acts in concert with the long thought causative vWDII mutation to cause the bleeding disorder in the Pointer breeds. Type III is rare in dogs as well and only found in Scottish Terriers [7, 8], Shetland Sheepdogs [7, 9], Kooikerhondje [10, 11] and Chesapeake Bay Retriever [12]. This is the most severe form of vWD with no detectable amount of vWF in plasma [13].

The causal mutation for vWDI was originally described in the Doberman Pinscher and is caused by a G > A (OMIA ref.: [14]) transversion in the last nucleotide of exon 43 coding for von Willebrand factor [3, 15]. This base substitution activates a cryptic splice site just few nucleotides upstream of the normal splice site. The resulting frame shift leads to the formation of a truncated protein of 119 amino acids [15]. The same mutation was also found in various other breeds [16]. In most of these breeds inheritance of vWDI was described as an autosomal recessive trait unlike the human disorder, which is inherited in an autosomal dominant manner with incomplete penetrance.

**Fig. 1:**
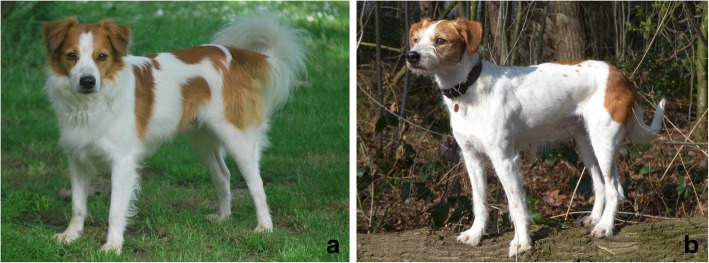
Purebred Kromfohrländer. Kromfohrländer come in two different coat types, smooth haired, with a smooth face, no beard and long soft hair, while the wirehaired Kromfohrländer have a beard. A smooth haired Kromfohrländer is shown in **a** and a wirehaired representative in **b**

The Kromfohrländer (Fig. 1a and b) was established in the years after World War II in Germany based on two founders dogs, most likely a Griffon Vendéen and a Fox Terrier but the exact breeds remain unclear. From these progenies the Kromfohrländer was bred and 10 years later in 1955 was first recognized by Fédération Cynologique Internationale (FCI). The Kromfohrländer is an exceptionally rare breed with a small number of registered dogs in different stud books. In 2018, 7 dogs of this breed were registered in Austria and 229 in Germany. Based on a small number of founders and the early recognition by the FCI resulting in closed studbooks, the inbreeding level of the Kromfohrländer is very high. The small population size for more than 50 years hasn’t spared the breed from disorders like epilepsy, hyperkeratosis, hereditary cataracts, cystinuria and autoimmune disorders like immune mediated hemolytic anemia or polyarthritis. Some of these disorders like hereditary footpad hyperkeratosis [17] or hyperuricosuria [18] can be genetically tested.

To avoid a further increase in disorders and improve the health situation as well as genetic diversity within the Kromfohrländer, a cross breeding program with Dansk-Svensk Gårdshund (Danish–Swedish Farmdog) was initiated from the breeding club ProKromfohrländer e. V. in Germany. Health and genetic monitoring of Kromfohrländer which is part of this breeding program showed a high number of dogs being heterozygous and homozygous for the vWDI mutation. Reports on excessive bleeding during surgeries, birth or neutering/spaying in the past are well known but without any clear association to a vWDI bleeding disorder.

The purpose of this study was to investigate if the G > A mutation in the *VWF* gene known and firstly reported from the Doberman Pinscher also leads to typical vWDI symptoms in Kromfohrländer dogs due to a reduced vWF serum concentration in both, heterozygotes and homozygotes. Moreover, we wanted to test the hypothesis that vWDI in the Kromfohrländer is unlike most other breeds in that it is inherited as an autosomal dominant trait with an incomplete penetrance comparable to the mode of inheritance in human vWDI and establish a causal link between reports of excessive bleeding and the disorder.

## Material & methods

Study Population – Purebred Kromfohrländer as well as F1, F2 and F3 siblings from outcross matings with Dansk-Svensk Gårdshund were included in this study. Dogs of different ages were recruited through ProKromfohrländer e.V. Germany or dog owners who voluntary supported the study. In cases for which there was no genetic testing result for vWDI, dog owners were asked to submit buccal swabs for performing genetic vWDI testing. Sample material was collected from all untested and available Kromfohrländer however with a special interest in those dogs that already showed typical signs of bleeding episodes.

### Medical history questionnaire

All study participants received a survey questionnaire to quantify the tendency of bleeding for each dog. Questionnaires were mailed to breeders or dog owners by a member of ProKromfohrländer e. V. Germany. The survey covered questions about haemorrhage, surgeries, spontaneous bleeding tendency in daily life as well during heat. Clinical data were submitted for all study dogs with an exception of 3 participants.

### Sample handling and preparation

Samples for DNA testing were non-invasively collected using buccal swabs. Sampling was performed by dog owners according to a detailed instruction. After taking mucosa samples from the muzzle, swabs were dried and sent back for determination of the genetic vWDI status. DNA from mucosa cells was purified with the automated DNA extraction system EZ1 advanced XL (Qiagen, Hilden, Germany) using the Investigator kit (Qiagen, Hilden, Germany) according to the manufacturers manual. DNA was quantified by using the Quantus™ Fluorimeter (Promega, Mannheim, Germany) and the QuantiFluor dsDNA System (Promega, Mannheim, Germany). DNA samples were diluted to a final concentration of 2 ng.μl^− 1^ with nuclease free water for subsequent genotyping PCR.

### Plasma von Willebrand factor and vWDI genotyping

Results of % blood vWF level were forwarded to our laboratory on dog owner’s initiative or ProKromfohrländer e.V. Germany. The gene variant of vWDI c.7437G > A was determined by KASP genotyping according to the manufacturers manual (LGC Genomics Ltd., UK). The assay was designed against the vWDI mutation within a 50 base pair upstream and downstream region of the G > A mutation. The WT allele was labelled with FAM and the vWDI associated allele with HEX. Amplification reactions contained 5 μl of extracted gDNA (2 ng.μl^− 1^) in a total reaction volume of 10 μl, complemented with 2x KASP master mix (Standard Rox, LGC Genomics Ltd., UK). Cycling conditions were carried out according to the manufacturer’s specifications using a Roche 480 Light Cycler (Roche, Basel, Switzerland). Samples of study dogs were used in replicates for a total of two genotyping PCRs, according to the standards and guidelines for clinical canine genetic testing laboratories [19]. For evaluation, endpoint genotyping was used to distinguish three possible cluster groups referring to the genotypes: WT/WT = c.7437G; WT/MUT = c.7437G + c.7437A; MUT/MUT = c.7437A. For each run negative controls (NT) without template consisting of 5 μl KASP master mix supplemented with nuclease free water were used.

## Results

### Study population

87 Kromfohrländer composed of 62% females and 38% males including purebred Kromfohrländer, as well as F1, F2 and F3 so called project dogs from outcross matings with Dansk-Svensk Gårdshund (Danish–Swedish Farmdog) as part of the outcross program in the ProKromfohrländer e. V. Germany were included in this study. Of all study dogs 73.5% were purebred Kromfohrländer, 2.4% were F1 offspring from Kromfohrländer x Dansk-Svensk Gårdshund crosses, 18.3% were F2 offspring from F1 mating with a purebred Kromfohrländer and 5.8% were F3 offspring from F2 mating with a purebred Kromfohrländer (Table 1). The age span of all dogs was between 0.7 and 15 years with a median age of 3 years and an average age of 5 years. The biggest proportion, 44.8%, was between 1 to 4 years followed by 20.7 and 18.4% between 4 to 8 years and 8 to 12 years. About 9.2% were below one year and 6.9% older than 12 years.

**Table 1:** Distribution of study dogs based on breed status and genotype

	WT/WT	WT/MUT	MUT/MUT	Total
Purebred	14.9%	47.1%	11.5%	73.5%
F1 offspring	1.2%	1.2%	0.0%	2.4%
F2 offspring	2.3%	13.7%	2.3%	18.3%
F3 offspring	1.2%	3.4%	1.2%	5.8%

Purebred Kromfohrländer as well as different offspring generations from a cross breeding program with Dansk-Svensk Gårdshund (Danish–Swedish Farmdog) where included in this study. The distribution based on the breed status and the corresponding genotype (wild-type (WT/WT), heterozygous (WT/MUT), homozygous (MUT/MUT)) is shown in this table. With 73.5% most study dogs where purebred Kromfohrländer followed by 18.3% F2 offspring. Genotype distribution shows a clear shift to heterozygotes (WT/MUT) within all 4 breed states

### vWDI genotyping results

**Fig. 2:**
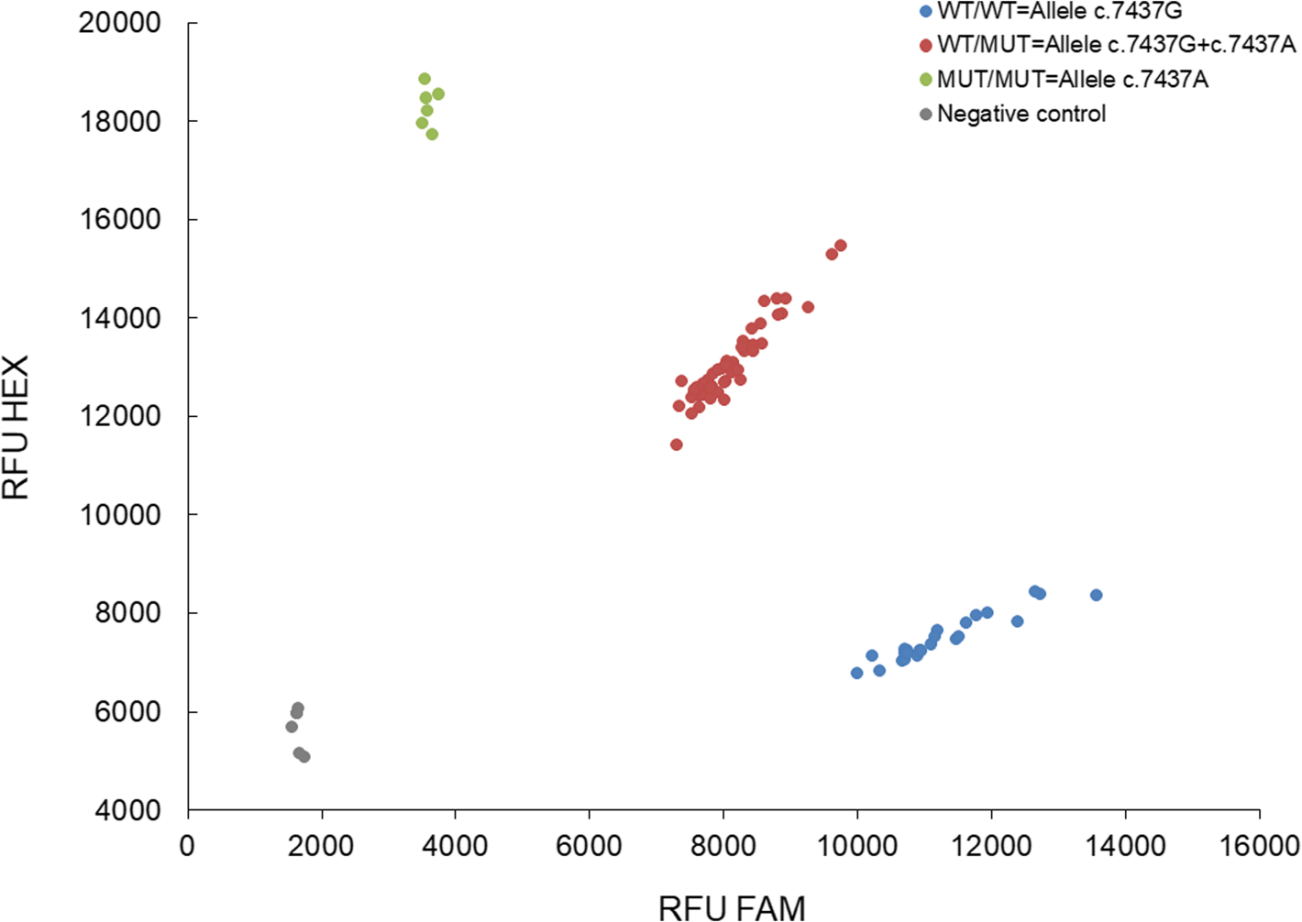
Example of vWDI genotyping in Kromfohrländer. Endpoint genotyping against c.7437G > A was used to identify the genotypes. Blue cluster = wild-type (WT/WT) with allele c.7437G, red cluster = heterozygous (WT/MUT) with both alleles (WT) c.7437G + mutated (MUT) allele c.7437A, green cluster = homozygous (MUT/MUT) with the vWDI related allele c.7437A. Controls of known genotypes were used in replicates for each cluster. Negative controls without template are shown in grey

**Fig. 3:**
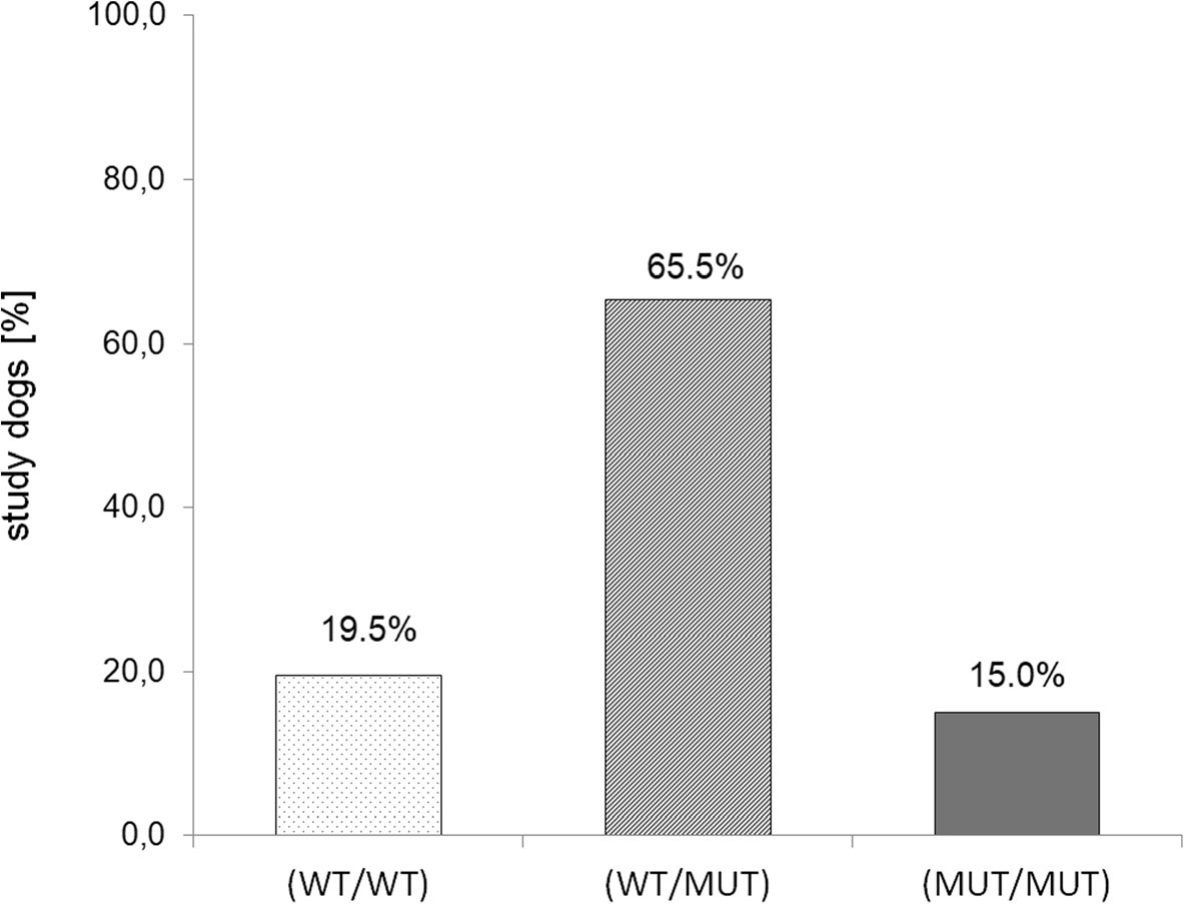
Distribution of von Willebrand type I genotypes in the Kromfohrländer study population. Based on genotyping results study dogs were categorized into the following three groups: wild-type (WT/WT), heterozygous (WT/MUT) and homozygous (MUT/MUT)

For genetic testing, study dogs were categorized in three different groups, wild-type (WT/WT), heterozygous (WT/MUT) and homozygous (MUT/MUT) for the vWDI mutation. Based on the genotyping data for the G > A transversion in the last nucleotide of exon 43 coding for von Willebrand factor (Fig. 2), 19.5% were wild-type (WT/WT), 65.5% were heterozygous (WT/MUT) and 15.0% were homozygous (MUT/MUT) (Fig. 3)*.* The frequency of the mutated allele within the study population is 48%. Within the group of purebred Kromfohrländer, 14.9% were wild-type (WT/WT), 47.1% were heterozygous (WT/MUT) and 11.5% were homozygous (MUT/MUT) for the vWDI mutation. In the F1offspring from outcross mating only wild-type or heterozygotes were detected, while in the F2 and F3 offspring all three genotypes (wild-type, heterozygous and homozygous) were present (Table 1).

### Bleeding tendencies

Information on bleeding tendencies was based on owner questionnaires which were provided for all study dogs with an exception of 3 dogs. Questions were primarily related to preceding surgeries or prolonged bleeding times for example during second dentition, heat, birth, implantations for chemical castration, injuries, or prolonged bleeding time after vaccination, as well as bruising and spontaneous epistaxis.

Of all tested Kromfohrländer, 21% showed bleeding symptoms compared to 76% of dogs without clinical signs up to the time the data were collected. For 3% no information about bleeding symptoms was available. In the case of two study dogs a definite classification was difficult. Nevertheless, based on the following description of symptoms we decided to assign both to the symptomatic group of dogs. One dog tested heterozygous (WT/MUT) for the vWDI mutation showed prolonged bleeding times, poor wound healing and encrusted pinna-rims. The second dog, tested homozygous (MUT/MUT), had a severely bleeding wound on his leg. It was not fully clear whether the severity of the injury caused the excessive bleeding or if there was indeed a direct association with von Willebrand’s symptoms because during a previous c-section this bitch was asymptomatic.

The most commonly reported symptoms in affected dogs were an excessive and prolonged bleeding during heat, prolonged bleeding times and hematomas after injuries, and massive hematomas and bleeding after castration in males. In the course of surgery, severe difficulties to stop bleeding were noted. After surgery, clinically affected dogs developed large area hematomas due to haemorrhages. In addition excessive bleeding during second dentition and gingival bleeding were observed. Hematomas also occurred after taking blood or implantations for chemical castration. Spontaneous epistaxis was also reported from several study dogs.

**Fig. 4:**
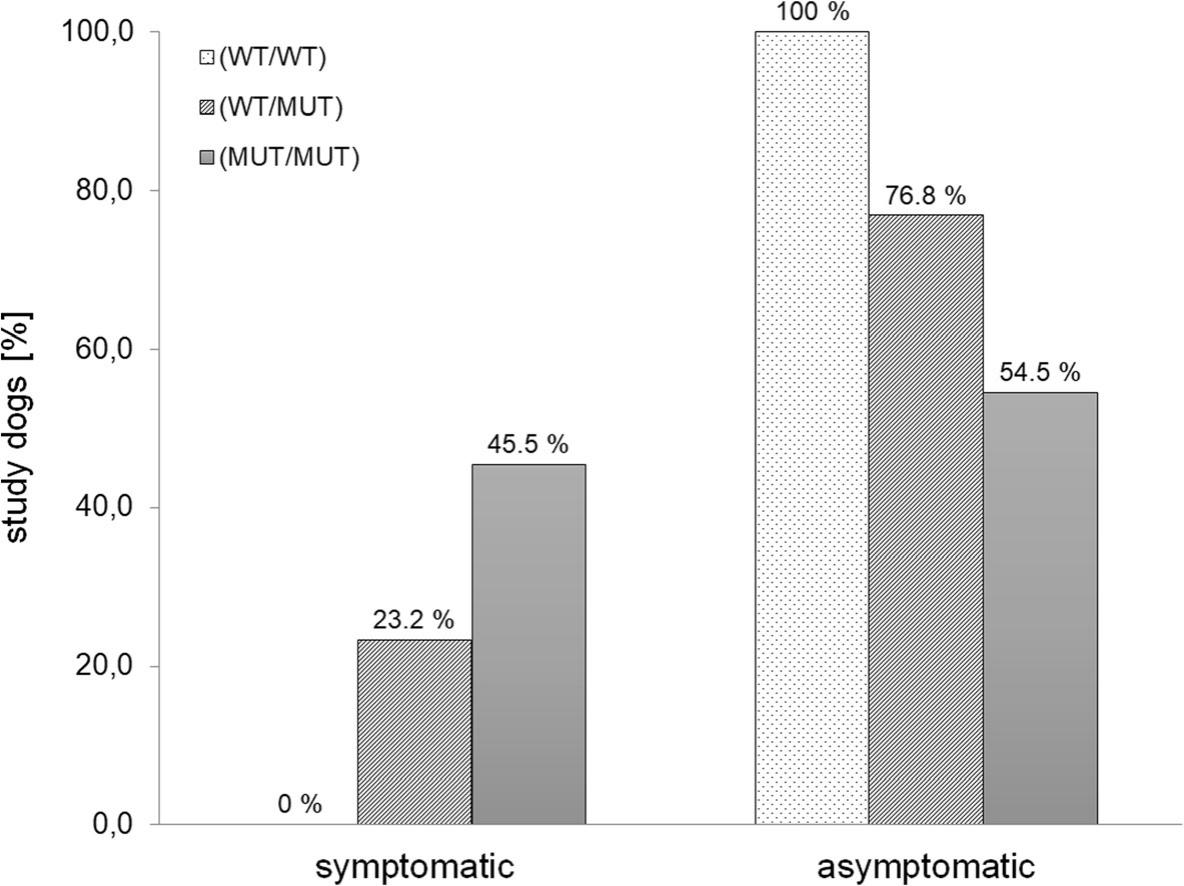
Distribution of von Willebrand type I symptoms in correlation with the corresponding genotype. Percentages of dogs with (symptomatic) or without symptoms (asymptomatic) were determined for each genotype group. 23.2% of heterozygotes (WT/MUT) showed bleeding symptoms compared to 45.5% of homozygous dogs (MUT/MUT). No symptoms were reported for wild-type (WT/WT) dogs without the mutation. Based on these results, hereditary of vWDI in Kromfohrländer seems to follow an autosomal dominant inheritance with an incomplete penetrance

Based on the information obtained from owner questionnaires the distribution of symptomatic and asymptomatic dogs within the different genotype groups was determined (Fig. 4). Dogs without available clinical data were excluded from this calculation. Within the wild-type group (WT/WT) of dogs tested for the vWDI mutation, none showed symptoms related to von Willebrand disease. Within the group of heterozygous vWDI dogs (WT/MUT) 76.8% were asymptomatic while 23.2% showed symptoms of von Willebrand syndrome. Among the dogs homozygous for the disease causing mutation (MUT/MUT) 45.5% showed symptoms, while 54.5% were asymptomatic up to the time of data collection. Kromfohrländer homozygous for the vWDI mutation had an OR 2.76 (95% CI 0.72 to 10.52, *P* = 0.1378) compared to those being heterozygous. The gender distribution within the group of dogs showing clinical symptoms is 56% bitches and 44% males.

### vWF serum concentration

**Fig. 5:**
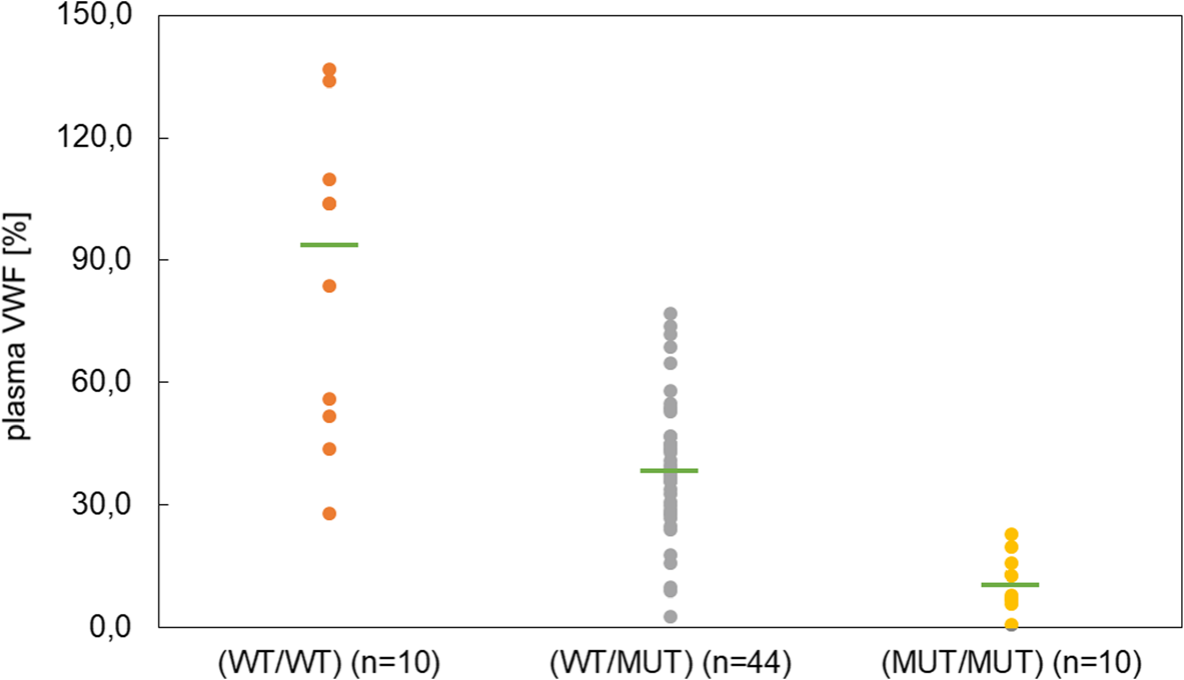
Correlation of plasma von Willebrand factor with the corresponding genotype in the Kromfohrländer breed. Von Willebrand factor and the three different genotypes (wild-type (WT/WT), heterozygous (WT/MUT), homozygous (MUT/MUT)) are shown for 64 Kromfohrländer dogs. Median is indicated in green and for wild-type 94.0%, for heterozygous 38.5% and for homozygous 10.5%. Significance between plasma vWF and the different genotypes was determined by a Kruskal-Wallis test (H = 29.5388; *p*-value < 0.00001; df = 2). The result is significant at *p* < 0.05

In addition to the genetic status of vWDI, von Willebrand factor serum levels were determined during health monitoring and results were forwarded by ProKromfohrländer e.V. or by dog owners or breeders to our laboratory. vWF serum levels were available from 64 dogs out of 87. The vWDI concentration in wild-type dogs (WT/WT) ranged from 28 to 137%. For heterozygous dogs (WT/MUT) the range was between 3 and 77%. For dogs homozygous (MUT/MUT) for the vWDI mutation the measured concentration was between 1 and 23%. The median vWF value for wild-type dogs was 94.0%, 38.5% for heterozygotes (WT/MUT) and 10.5% for homozygotes (MUT/MUT), respectively (Fig. 5). Age of first signs of bleeding symptoms was between 4 months and 12 years with a median age of 3 years. Males showed first signs of symptoms between 1 year and 12 years with a median age of 3.3 years. In females first signs were noticed with an age of 4 months during second dentation and 8 years. The median age of female dogs was slightly lower with a value of 2.5 years compared to males with 3.3 years.

## Discussion

The Kromfohrländer breed was established in the years after World War II based on two single founders dogs, most likely a Griffon Vendéen and a Fox Terrier. Due to the limited number of founders it is supposed that there is a high level of inbreeding within the Kromfohrländer population. A correlation between high inbreeding levels as well as a decreased genetic diversity with inherited and autoimmune disorders is widely discussed in the scientific community. Diseases such as autoimmune hemolytic anemia or polyarthritis are reported in the Kromfohrländer breed. In addition to autoimmune diseases different types of inherited disorders with known causative mutations like hereditary footpad hyperkeratosis [17] or hyperuricosuria [18] have been described in the Kromfohrländer breed. Genetic screenings [16] for known inherited disorders performed for health monitoring by ProKromfohrländer e. V. revealed a very high allele frequency for the vWDI mutation which was first described in Doberman Pinscher [3, 15] and recently reported in several other dog breeds [16].

In most breeds, von Willebrand disease type I is described as an autosomal recessive bleeding disorder. In the Kromfohrländer, vWDI seems to show an autosomal dominant mode of inheritance. Patients with a type 1 disease typically show reduced levels of von Willebrand factor plasma concentration. In the present study, 87 dogs were genotyped for the known vWDI mutation. 65.5% of all study dogs were heterozygous for the vWDI mutation. From 64 participants genetic data were compared with the vWF levels obtained from blood diagnostics. Within the study population heterozygotes had a median vWF serum concentrations of 38.5%. Dogs homozygous for the vWDI mutation showed a median serum concentration of 10.5% vWF. Therefore they carry a high risk for developing symptoms of a blood coagulation disorder due to the reduced factor concentrations. In general, dogs with vWF concentrations lower than 50% are classified as “at risk” and may have bleeding episodes. Oral mucosa with gingival bleeding, epistaxis and hematuria are known clinical signs associated with vWDI as a result of the decrease in platelet function. Injuries, trauma or surgery can lead to prolonged bleeding. It is also known that not all animals having a decreased vWF level exhibit an increased tendency to bleed. Based on genotyping data and vWF serum concentrations, a direct association between the occurrence of the mutation and a reduction in serum factor concentration can be postulated. While those dogs tested as vWF wild-type have a median vWF of 94.0%, this level decreases significantly depending on whether the dog is heterozygous or homozygous for the mutation. It is obvious that such a reduction has a negative effect on blood clotting.

vWF serum levels varied significantly within each group. Dogs genetically tested as heterozygous showed vWF levels ranging from 3% up to 77%. Comparable observations were also made in dogs homozygous for the vWDI mutations ranging from 1% up to 23%. Those dogs without any mutation showed levels between 28 and 137%. Alterations in the plasma vWF level can be due to different causes. It is well known that ageing or repeated freezing and thawing during prolonged transport to the diagnostic laboratory may affect von Willebrand levels in blood samples causing decreased factor levels and subsequently a false interpretation of the results [20]. Furthermore vWF can be influenced by inflammatory conditions, hormones, medication, pregnancy, heat or lactation. In addition the plasma concentration of individual dogs varies daily and weekly and this fact may be of clinical relevance. We can’t exclude that such factors had an influence on vWF levels, leading to the variations within the three different genotype groups. Nonetheless, a genotype-dependent reduction in serum factor levels of heterozygous and homozygous dogs could be clearly shown.

The genetic status of a dog with regards to vWDI may be predicted based on the vWF plasma concentration. Dogs being heterozygous for the vWDI mutation are usually having less than half of the normal vWF plasma concentration while homozygous dogs are presented with little or no vWF protein. Von Willebrand factor concentrations between 50 and 70% do not allow an accurate prediction of the genetic status. Values in this range can be the result of a homozygote wild-type but also of a heterozygous dog of the vWDI mutation as we have also shown in this study. A similar overlap is also found in lower ranges of vWF concentrations when discriminating between heterozygotes and homozygotes.

In order to prevent misinterpretation of vWF plasma concentrations, genetic testing should be carried out. Based on the genotyping results mating of dogs heterozygous or homozygous for vWDI can be avoided.

**Fig. 6:**
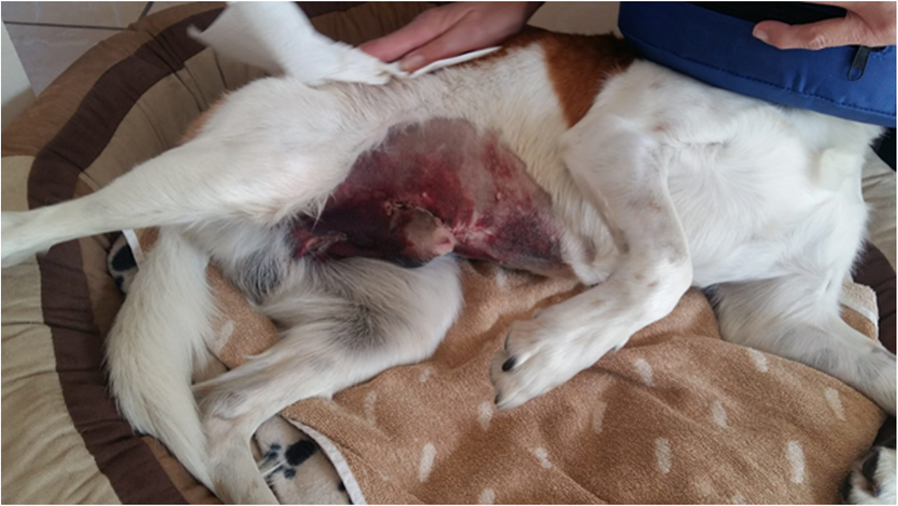
Kromfohrländer with excessive hematomas. This picture shows a male Kromfohrländer three days after neutering and surgical excision of cystine crystals and calculi due to cystinuria with massive hematomas. The dog is heterozygous (WT/MUT) for the von Willebrand type 1 mutation. The vWF serum level was determined with a value of 72%

An example of a heterozygous dog with a vWF plasma concentration of 72% is shown in Fig. 6. This purebred male Kromfohrländer showed massive bruising after neutering and excision of crystals due to an inherited form of cystinuria. According to owner completed questionnaires excessive bleeding during and after surgeries or giving birth was also observed in other Kromfohrländer. Typical symptoms of bleeding were not only observed in dogs homozygous (MUT/MUT) for the vWDI mutation but also in heterozygotes (WT/MUT). This is supported by the fact that in 23.2% of all heterozygotes symptoms were reported compared to 45.5% of all homozygotes while all wild-type tested dogs where asymptomatic. Based on these results, heredity of von Willebrand disease type I in the Kromfohrländer breed seems to follow an autosomal dominant inheritance with incomplete penetrance. The risk for developing symptoms increases with the number of mutated alleles and an odds ratio (OR) of a homozygous dog is 2.76 compared to a heterozygous dog. The existence of a possible second mutation linked to and acting in concert with the known causative vWDI mutation can not be excluded at the moment and might be one reason for an incomplete penetrance. Furthermore, the blood clotting cascade involves 13 different factors. Each one of these factors could have variation that influences coagulation. Thus, it is not surprising with such a complex process that variable expressivity and incomplete penetrance is present. It can be challenging to get the dog owners and breeders to understand these genetic complexities.

Due to the very high number of heterozygous dogs in the population long term breeding strategies should be realized to minimize the frequency of the vWDI allele in the breeding stock and to avoid a further distribution in the following generations of Kromfohrländer. It is important to note that such breeding strategies should not lead to a further reduction of genetic diversity and an additional increase in the inbreeding level. Based on the fact that the risk of developing bleeding symptoms is higher in homozygous (MUT/MUT) dogs than in heterozygotes (WT/MUT) we strongly recommend mandatory genetic testing of vWDI. If the vWDI genotype of stud dogs was determined, mating can be planned in a way to avoid homozygous offspring from breeding. The high allele frequency of 48% and the limited number of stud dogs within the Kromfohrländer population will need a stepwise reduction of the frequency over generations.

## Conclusion

Von Willebrand type I mutation has been reported in a variety of breeds. Based on our results with an allele frequency of 48%, the Kromfohrländer breed is now one of them.

A comparison of the genotyping data with von Willebrand factor serum concentrations confirms a causal relationship between the occurrence of this mutation and a reduction of the serum factor level. Depending on the genotype (wild-type, heterozygous or homozygous), plasma vWF concentrations vary, leading to an increasing risk of developing bleeding symptoms with decreasing serum factor concentrations. In the case of vWD type I in the Kromfohrländer breed, it can be assumed that inheritance follows an autosomal dominant manner with incomplete penetrance, since both heterozygous as well as homozygous dogs developed classical symptoms of this coagulation disorder. To avoid further distribution of the mutant allele within the population it would be of importance to perform genetic testing of vWDI and realize a long term breeding program for reducing the allele frequency.

## Abbreviations

MUT: Mutated
vWDI: Von Willebrand disease type I
vWF: Von Willebrand factor
WT: Wild-type
